# Assessment of molecular modulation by multifrequency electromagnetic pulses to preferably eradicate tumorigenic cells

**DOI:** 10.1038/s41598-024-81171-x

**Published:** 2024-12-03

**Authors:** Roberta Piredda, Luis G. Rodríguez Martínez, Konstantinos Stamatakis, Jorge Martinez-Ortega, Alejandro López Ferráz, José M. Almendral, Yolanda Revilla

**Affiliations:** 1 Centro de Biología Molecular Severo Ochoa (CSIC-UAM), Universidad Autónoma de Madrid, Cantoblanco, Madrid, 28049 Spain; 2 Universidad de Sancti Spíritus, Sancti Spíritus, Cuba; 3 Universidad de La Laguna, Santa Cruz de Tenerife, Spain; 4 IRYCIS, Madrid, Spain

**Keywords:** Biophysical methods, Cancer metabolism, Cell biology

## Abstract

Physics methods of cancer therapy are extensively used in clinical practice, but they are invasive and often confront undesired side effects. A fully new equipment that allows sustained emission of intense and time-controlled non-ionizing multifrequency electromagnetic pulse (MEMP), has been applied to eukaryotic cells in culture. The equipment discriminates the overall electronegative charge of the cell cultures, and its subsequent proportional emission may thereby become higher and lethal to cancer cells of generally high metabolic activity. In contrast, low tumorigenic cells would be much less affected. We tested the specificity and efficacy of the equipment against a collection of (i) highly tumorigenic cells of human (glioblastoma, cervical carcinoma, and skin) and mouse (colon adenocarcinoma) origin; (ii) cell lines of much lower tumorigenicity (non-human primate kidney and mouse fibroblasts), and (iii) primary porcine macrophages lacking tumorigenicity. Time and intensity control of the MEMP allowed progressive decay of viability fairly correlating to cell tumorigenicity, which was provoked by a proportional alteration of the cytoplasmic membrane permeability, cell cycle arrest at G2, and general collapse of the actin cytoskeleton to the perinuclear region. Correspondingly, these effects drastically inhibited the proliferative capacity of the most tumorigenic cells in clonogenic assays. Moreover, MEMP suppressed in a dose-dependent manner the tumorigenicity of retrovirally transduced luciferase expressing colon adenocarcinoma cells in xenografted immune-competent mice, as determined by tumor growth in a bioluminescence imaging system. Our results support MEMP as an anti-cancer non-invasive physical treatment of substantial specificity for tumorigenic cells with promising therapeutic potential in oncology.

**Supplementary Information:**

The online version contains supplementary material available at 10.1038/s41598-024-81171-x.

## Introduction

Low-frequency magnetic fields (LMF) are experimentally used to treat tumors, since they induce nonionizing, non-thermal, and noninvasive effects on tissues, inhibiting the proliferation of tumor^1–3^. Other in vitro experiments have shown that LMF may limit the growth of tumor cells^4,5^, whereas non-tumoral cells do not suffer major alterations^6^.

The mechanisms by which LMF exerts these effects have been only partially addressed. For example, it has been suggested that LMF affects the cytoplasmic membrane of the tumoral cells, and some apoptotic events have also been shown to be involved^7^. Other studies proposed that the induction of reactive oxygen species (ROS) correlates to the inhibitory effect of LMF, and that the intercellular environment and intercellular aggregation are necessary events for magnetic inhibition. In a recent report^8^, a fixed magnetic field of 5 mT and 20 Hz was used being the magnetic field generator in direct contact with cells, inducing antiproliferative effects in the tumor cells studied.

The equipment used in this report, developed by PASO ALTO BIOTECHNOLOGY INC, consists of a new LM non-ionizing radiation technology that enables the sustained delivery of intense, time-controlled, multi-frequency electromagnetic pulses (MEMP), which can be applied to cells in culture. Major features of the modulator equipment are further explained in the “Materials and methods” section, being a new device that discriminates the overall electronegative charge of cell cultures. As cancer cell metabolism may impose drastic differences in their electronegativity^9^, it was of paramount interest to explore whether the modulator equipment can differently impact cells at distinct tumorigenicity stages.

The differences in the observed responses between cancerous and non-cancerous cells are explained by the intrinsic electrical characteristics of each cell type. Although the device does not exert a direct and selective effect on a particular cell type, the electrical peculiarities of cancer cells induce a differentiated response compared to normal cells to the specific electromagnetic stimulus generated. The main electrical properties of the cells involved in this phenomenon might include: depolarization of the membrane potential, dysfunction of ion channels, alteration of electrical conductance, modification of cell membrane composition and alteration of intercellular communication^10–12^.

Therefore, the MEMP treatment effects on relevant parameters of cell biology were tested in a collection of mammalian cell lines with diverse tumorigenicity. For this, we have investigated the effects of this technology on cell viability, cell cycle progression, and clonogenic capacity, of cells with diverse degree of tumorigenicity. Importantly, among the potential targets of the MEMP treatment that could account for the cellular responses, we focused in the actin cytoskeleton as electromagnetic fields may disrupt cytoskeletal organizations at multiple levels^13–15^. Thus, we addressed a quantitative confocal IF analysis of the actin cytoskeleton before and immediately post- treatments of cells with distinct tumorigenicity. Finally, attempting to further support the relevance of the MEMP treatment for future in vivo applications, the study was extended to investigate the in vivo tumor-forming ability of colon cancer cells subjected to a single MEMP treatment. This aim was supported by a preliminary assessment of the effect of the MEMP treatment in the animal’s well-being in order to ascertain the safety of the treatment. Our study collectively supports the therapeutic potential and tumor specificity of MEMP that could be translated into clinical practice.

## Materials and methods

### Cells

Cell lines were cultivated in Dulbecco’s Modified Eagle Medium (DMEM) supplemented with 2 mM l-glutamine, 0.4 mM non-essential amino acids,100 U/mL gentamicin, and 5 or 10% fetal calf serum (FBS; Invitrogen Life Technologies). All cells were grown at 37 °C with 5% of CO_2_ in a humidified air (95%). The origin of our cell lines was as follows: COS-1 (CRL-1650), CV-1 cells (CCL-70) from African green monkey kidney, HEK-293T cells (CRL-3216) (Human embryonic kidney cells), and A9 mouse fibroblasts (CRL-3265), were obtained from the American Type Culture Collection (ATCC); U373 MG (HTB-17) human glioblastoma astrocytoma was purchased from ATCC; the MC-38-Luc cells, derived from MC-38 cell line (SCC172, Sigma-Aldrich), C57BL/6 murine colon adenocarcinoma cells and stably expressing luciferase gene, were generated in our laboratory as described below; HeLa cells (CCL-2) from human cervical carcinoma were obtained from the ATCC; and the HaCaT human keratinocyte cell line was kindly provided by Dr. Miguel Quintanilla (Instituto de Investigaciones Biomédicas “Alberto Sols”, Madrid, Spain). Porcine alveolar macrophages (PAM) were obtained by a bronchoalveolar lavage as previously described^16^, and cultivated in DMEM supplemented with 10% porcine serum.

### Vectors and transfection

To obtain MC-38 cells constitutively expressing the bioluminescence marker luciferase, the pLVX-IRES-Luc lentiviral vector was constructed. To this aim, we cloned the luciferase gene from the mammalian pIRES-Luc vector, provided by Encarna, into a pLVX-puro empty vector. For pLVX-IRES-Luc cloning, we used 2X Phusion Maser Mix HF (Thermo Scientific) and In-Fusion technology (Takara) by using the following oligo probes:

5’TCGCTAGCGCTACCGGACTCAGCAGGTTTCCCCAACT3’and 5’TAGAATTATCTAGAGTCGCGttacacggcgatctttccgccc3’ to amplify the luciferase gene, and 5’GAGTCCGGTAGCGCTAGC3’ and 5’CGCGACTCTAGATAATT3’ to linearize the pLVX-puro vector and insert the amplification. For the transfection protocol, FuGene HD transfection reagent (Promega) was employed, following the manufacturer’s instructions.

### Cells transduction

The lentivirus was grown in HEK-293T packaging cell lines. Supernatants were harvested and virus luc-transducing was used to inoculate MC-38 cells. Transduced cells were selected with puromycin for 14 days, until the death of control cells. To investigate the stable expression of the lentiviral vector, cells were analyzed by Luc-Pair Duo Luciferase HS Assay Kit (Genecopoeia). Luciferase readings were performed using the FLUOstar OPTIMA reader (BMG LabTech).

### Molecular modulation

The modulator equipment was designed and developed by the North American corporation Paso Alto Biotechnology (Inc.), under industrial secret. It is made up of two fundamental structures, an electromagnetic induction system assisted by sensors, and a high-power hardware system. The modulator was used to irradiate the different cell line samples selected for this study with a powerful multifrequency electromagnetic field of non-ionizing emission (f < 30 Hz). The operational value of electromagnetic flux emitted by this device is greater than 2 Tesla (B > 2 T). All modulated cell lines were exposed to the MEMP treatment by seeding them individually in 12-well Falcon cell culture plates that were placed on the induction system by the addition of sensors per well. These sensors assisting the induction system are designed to determine the electrical permeability of the medium safely, and are responsible for sending the necessary operating signal to the switch circuit, which in turn transmits sufficient energy to the induction system to generate the electromagnetic field with the required characteristics. The electronic perturbation induced by this MEMP on the culture medium during the corresponding exposure times of 2.5 and 5 min (min) may alter the electronic distribution of the biomolecules in a cell-type-dependent manner.

### Viability determination by MTT

Upon the indicated MEMP treatments, 10^4^ cells/well were seeded in a 96 multi-well plate. After 24 h (h), the cell medium was removed, cells were washed with PBS, and the medium was replaced. When negative control cells (T0, non-modulated) reached the confluence (between 2 and 4 days), viable cell numbers were estimated by their mitochondrial activity. For this, 20 µl of 5 mg/ml MTT (3-(4,5-methylthiazol-2-yl)-2,5-diphenyltetrazolium bromide) were added directly to the medium and incubated at 37 °C in the dark for 4 h. Cells were then washed with PBS for 5 min under shaking in the dark, PBS was removed and 100 µl of dimethyl sulfoxide (DMSO) were added. Finally, after incubation for 30 min in the dark under shaking, the absorbance at 570 nm was measured with a spectrophotometric plate reader Claro Star System (BMG LabTech). All experiments were carried out in triplicates.

### Colony formation assay

Variable numbers of cells in the range of 10^3^ to 10^4^ either control or MEMP-treated were seeded in 60 mm dish plates in their corresponding culture medium with 10% FCS, and incubated at 37 °C for 2–3 weeks under medium replacement every 3 days. Once control cells formed visible colonies of significant size, the cells were washed in PBS and fixed in 4% formaldehyde for at least 2 h at room temperature (RT). Cells were then stained with 0,2% crystal violet diluted in 4% formaldehyde for 1 h at RT, washed in water, left to dry, and then the number of colonies for each plate was visually counted.

### Viability determination by cytometry

Control and MEMP-treated cells were collected immediately after the treatment, centrifuged at 1500 rpm for 5 min, and the supernatant was discarded. The cell pellet was washed twice in PBS and centrifuged at 2000 rpm for 4 min to remove the excess of serum. Cells were then suspended in 450 µl of PBS and stained with the Ghost Dye Red 780 (1ul/ml) (TONBO biosciences) for 5 min at 37 °C in the dark. Ghost Dye Red 780 is an amine-reactive dye able to discriminate viable from necrotic mammalian cells with compromised membrane functioning. Cells were fixed in 4% paraformaldehyde (PFA) for 10 min at RT and suspended in 200 µl of PBS-Staining containing 1% of bovine serum albumin (BSA). The cell viability was determined by a FACS Canto (BD bioscience) equipment. A number of 10,000 cellular events were analyzed using the Flow-jo I.6.5 software.

### Cell cycle analysis

Control and MEMP-treated cells were collected at 48 h after treatment, the pellet was washed in PBS, and cells were fixed with 1 ml of 70% ice-cold ethanol added in a drop by drop way using vortex, followed by an overnight incubation at -20 ºC. Fixed cells were centrifuged for 5 min at 1500 rpm and washed twice with PBS. The residual volume was shaken, and cell samples were labeled incubating with PI/RNase Staining Buffer (BD Pharmingen) for 30 min at RT in the dark. Cell cycle assay was performed by FACS Calibur (Becton Dickinson) and around 10,000 events for each sample were collected. Data were analyzed by Flow-jo I.6.5 software.

### Actin cytoskeleton analysis

Control and MEMP-treated cells were washed in PBS, fixed immediately after modulation with 4% PFA for 10 min at RT in the dark, and permeabilized in 0,1% Triton™ X-100 and PBS 1X. Cells were treated with blocking buffer (0,1% Triton™ X-100, 1% gelatin, in PBS 1X) for 1 h, washed twice, incubated with DAPI for 10 min, and then stained by phalloidin-Alexa 555 (Thermofisher; 1:500) diluted in binding buffer (1% gelatin in PBS 1X ) for 1 h at RT. Cells were finally washed twice with PBS and mounted in Mowiol® medium (Sigma-Aldrich). Images were taken by using confocal microscopy LSM800 coupled to an inverted Axio observer (Zeiss) with a 60x oil immersion objective lens. Cytoplasmic images were processed using a custom ImageJ macro and preprocessed by applying background subtraction followed by a Gaussian blur to reduce noise. For cytoplasmic segmentation, the Cellpose plugin was used with the cyto2 model^17^ and all the generated ROIs were filtered based on DAPI channel to ensure accuracy. Lastly, for each segmented region, the area was measured using ImageJ’s measurement tools.

### Xenograft cancer model

All animal care procedures used in this study were carried out in accordance with ARRIVE https://arriveguidelines.org/ guidelines. All animal procedures were performed in strict accordance with the European Commission legislation for the protection of animal use purposes (2010/63/EU). The protocol for the treatment of the animals was approved by the Comité de Ética de la Dirección General del Medio Ambiente de la Comunidad de Madrid, Spain (PROEX 217.4/23) and was supervised by the Ethics Committee of CBM (Madrid, Spain). Mice were purchased from Janvier Labs, Le Genest-Saint-Isle, France. Mice were housed under a 12-h light/dark cycle in a specific pathogen-free facility with controlled temperature and humidity (20–24 °C, 45–65% humidity) and allowed access to food and water ad libitum. Nineteen four-week-old female immunocompetent C57BL/6 mice (initial weight 17–21 g), were injected subcutaneously in the flank with 0.5 × 10^6^ colon mouse adenocarcinoma cells (MC-38-Luc). In vivo tumor cell bioluminescence was monitored and quantified using the IVIS Spectrum System (PerkinElmer) after IP injection of D-Luciferin (150 mg/kg), with the mice anesthetized with isoflurane inhalation (4% induction, 2% maintenance of anesthesia). Body weight and general physical status were recorded daily, and the mice were sacrificed by carbon dioxide (CO_2_) inhalation at the end of every experiment or when reaching the endpoint criteria.

### Tumor forming capacity of MEMP-treated cells

MC-38-Luc cancer cells were seeded in 12-well plates and modulated respectively for 1.5 and 2 min, based on previous viability experiments described above. It is important to note that the MEMP treatments were set at short times in order to collect intact cells that were inoculated into mice to test their tumor-forming capacity. After the MEMP treatment, the cells were centrifuged and resuspended in OptiMEM (Gibco) medium and counted to generate homogeneous suspensions of 0.5 × 10^7^ MC-38-Luc cells/ml. The C57BL/6 mice were injected with 100 µl of the cell suspension per animal corresponding to samples of control non-modulated cells, cells modulated for 1.5 min, and cells modulated for 2 min. The experiment was performed twice with independent groups of mice purchased weeks in between. After injection, mice were evaluated within the same day by IVIS Spectrum Imaging System (PerkinElmer) for luciferase bioluminescence as well at the time points indicated in Fig. 6 to determine the tumor growth generated by controls and modulated cells. The mice were anesthetized with isoflurane inhalation (4% induction, 2% maintenance of anesthesia). IVIS data were analyzed with Living Image 4.8.0 (PerkinElmer) evaluating the total radiance (photons/sec) of the tumors. The mice were sacrificed by carbon dioxide (CO_2_) inhalation at the end of every experiment or when reaching the endpoint criteria.

### Monitoring MEMP safety in mice

Four healthy C57BL/6 mice (initial weight 17–26 g), 2 females and 2 males, were subjected to a single MEMP treatment. The modulation was applied by placing the mice on the same structure (emitter focus) used to modulate the cells. For this, the mice were allocated in small methacrylate boxes which limited movement maintaining mice near the electromagnetic emitter. The parameters of the modulator configuration were kept unchanged, with respect to those used in all experiments with cells, which contemplate a frequency lower than 30 Hz and an electromagnetic field higher than 2 Tesla. Both immediately before and at 72 h post-treatment, the animals were weighed and subjected to blood parameters inspection as follows. Blood samples were collected from the submandibular vein and prepared for leukocyte counting in the presence of EDTA, and then diluted with 1% Turk’s solution (glacial acetic acid, in aqueous solution of gentian violet). Leucocytes were counted in triplicates using a Neubauer chamber under the microscope. Ten days post-treatment the mice were again weighed and inspected for leukocyte counts as above. Values between MEMP-treated and control untreated control mice were quantitatively compared. In addition, basic animal health parameters were visually recorded daily, such as appetite and the presence of side effects putatively caused by the treatment.

### Analysis of possible MEMP-induced temperature changes

Before and after the MEMP treatments, the temperature of the U373, HeLa, and MC-38-Luc tumor cells, was assessed using conventional thermometers introduced directly into each well of the 12-well Falcon plate undergoing modulation. Temperature was recorded in at least three independent modulations performed at several times (in the range of 0–5 min) for each tumor cell type.

## Results

### The MEMP treatment inhibits the viability of malignant cancer cells whereas low tumorigenic cells are not affected

To study whether the MEMP could impact mammalian cell physiology, we selected a collection of established cell lines with well-reported origin and tumorigenicity, as well as primary porcine macrophages (see Table 1), and compared their viability and growth features in response to a MEMP treatment applied at several times.

**Table 1:** Description of MEMP-treated cells.

Name^a^	Origin^b^	Malignancy^c^	References^d^
MC-38	Murine colon adenocarcinoma derived from primary induced tumors in different inbred strains of mice	High in nude and immunocompetent C57BL/6 mice	^18–20^
U373 MG	A human glioblastoma astrocytoma derived from a malignant tumor	High as measured in subcutaneously transplanted nude mice	^21,22^
HeLa	Human cervical carcinoma transformed by Papillomavirus HPV18	High as measured by tumor formation after subcutaneous transplantation in nude and other immunodeficient mouse strains.	^23–25^
HaCat	Human skin	High when injected subcutaneously into thymus aplastic nude mice (Swiss/c nu/nu)	^26–28^
CV-1	Kidney fibroblasts of a male adult African green monkey	Low but significant upon high passages number as tested in anti-thymocyte globulin (ATG) treated newborn Wistar rat	^29,30^
COS-1	CV-1 simian cells transformed with the early region of the polyomavirus SV40 genome	Unpublished	^31^
A9ouab^r^11	Mouse fibroblasts	Low and restricted to some clones. Measured in y-irradiated syngeneic newborn and nude mice.	^32–34^
PAM	Porcine alveolar macrophages isolated from the lungs	Null	^16^

^a^Common name.

^b^Origin and major features.

^c^Capacity to generate tumors in the outlined experimental animals.

^d^Primary description and malignancy-related references.

**Fig. 1:**
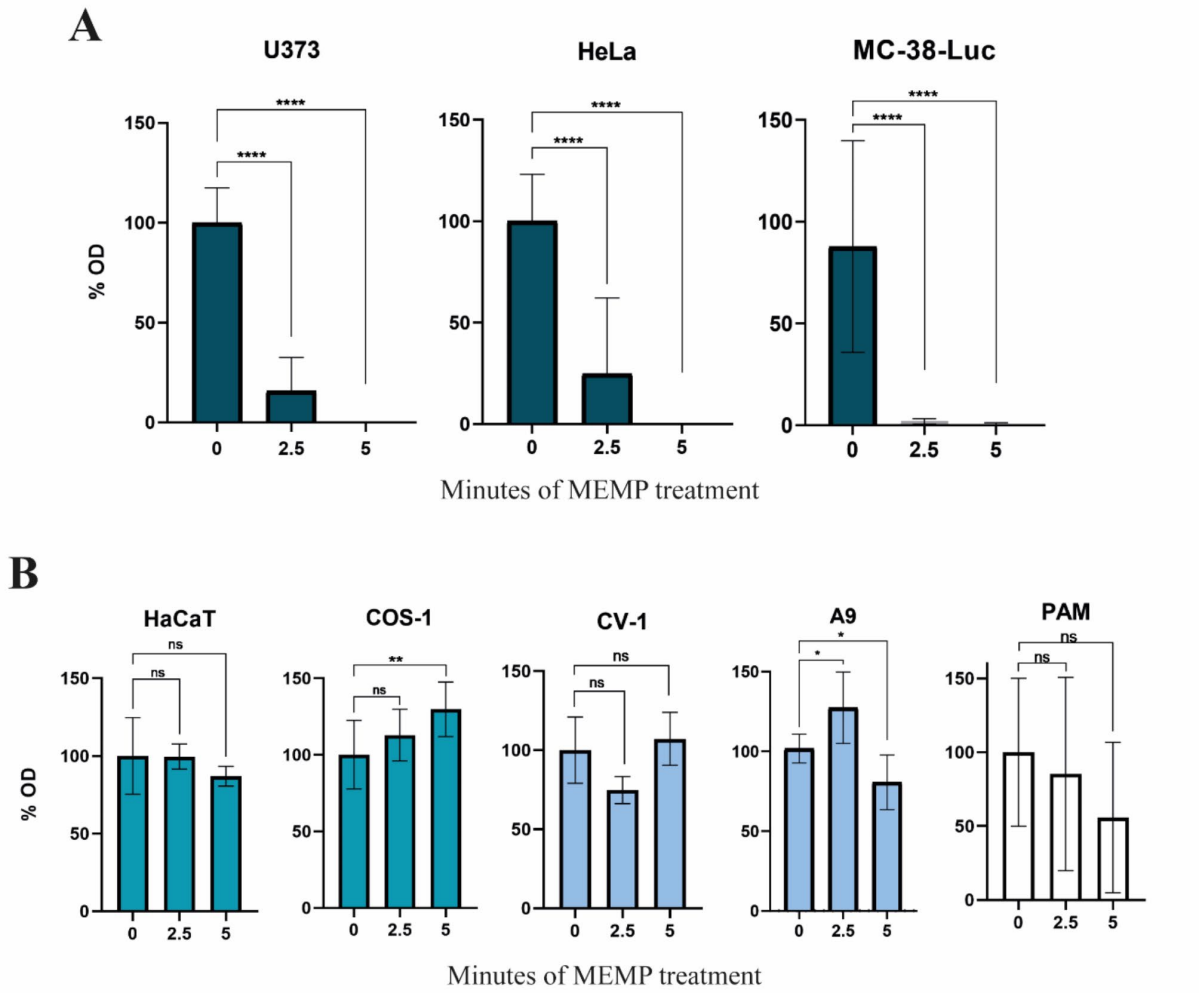
Degree of inhibition of cell viability caused by MEMP as evaluated by an MTT assay. Quantitative determination of cell metabolic activity by MTT in non-treated controls and upon the indicated MEMP treatments. (**A**) Evaluation of cell viability in U373 MG, HeLa, and MC-38-Luc cancer cell lines treated from 0 to 5 min. (**B**) Evaluation of viability after MEMP treatment applied for 2.5 and 5 min to HaCaT, CV-1, COS-1, A9 cell lines, and PAM. Data are the mean with standard errors obtained from triplicates and were statistically analyzed by using a one-way ANOVA with a Dunnett’s test (*P < 0.05; **P < 0.01; ****P < 0.0001).

Firstly, the U373 MG, HeLa, and MC-38-Luc highly malignant cell lines were modulated for 2.5, and 5 min in PBS, and cell viability after treatment was primarily evaluated by mitochondrial activity using the MTT colorimetric assay, a marker of cellular metabolic activity^35^, as described in Materials and Methods. The analysis shows a progressive effect of the treatment at 2.5 min of the modulation time, and no cell viability could be detected when either U373, HeLa, or MC-38-Luc cells were modulated for 5 min (Fig. 1A). In particular, MC-38-Luc seemed to be more sensitive to the treatments as compared to U373 and Hela cells since no viable cells were detected after 2.5 min of treatment. Of note, in order to evaluate putative temperature changes that could affect our analysis, control experiments performed with conventional thermometers (see Materials and Methods) showed a maximum variation of 0.4 °C (32.72 °F) regardless modulation time, which should be attributed to sample handling rather than a thermal effect induced by the electromagnetic exposure.

To further explore the susceptibility of mammalian cells to the MEMP treatment, low-tumorigenicity cells were also evaluated. For this, HaCaT, CV-1, COS-1, A9 cell lines, and importantly PAM primary macrophages, were subjected under identical seeding conditions in 12-multiwell dishes to the MEMP treatment at different time points. Figure 1B shows that the viability of these poorly tumorigenic cells was significantly non-affected by the modulation. Overall, the obtained data indicated very low sensitivity of the non and poorly tumorigenic cells to MEMP, whereas all the malignant cells analyzed were importantly affected by the treatment.

### The MEMP treatment inhibited the clonogenic capacity of malignant cancer cells but not that of poorly tumorigenic cells

**Fig. 2:**
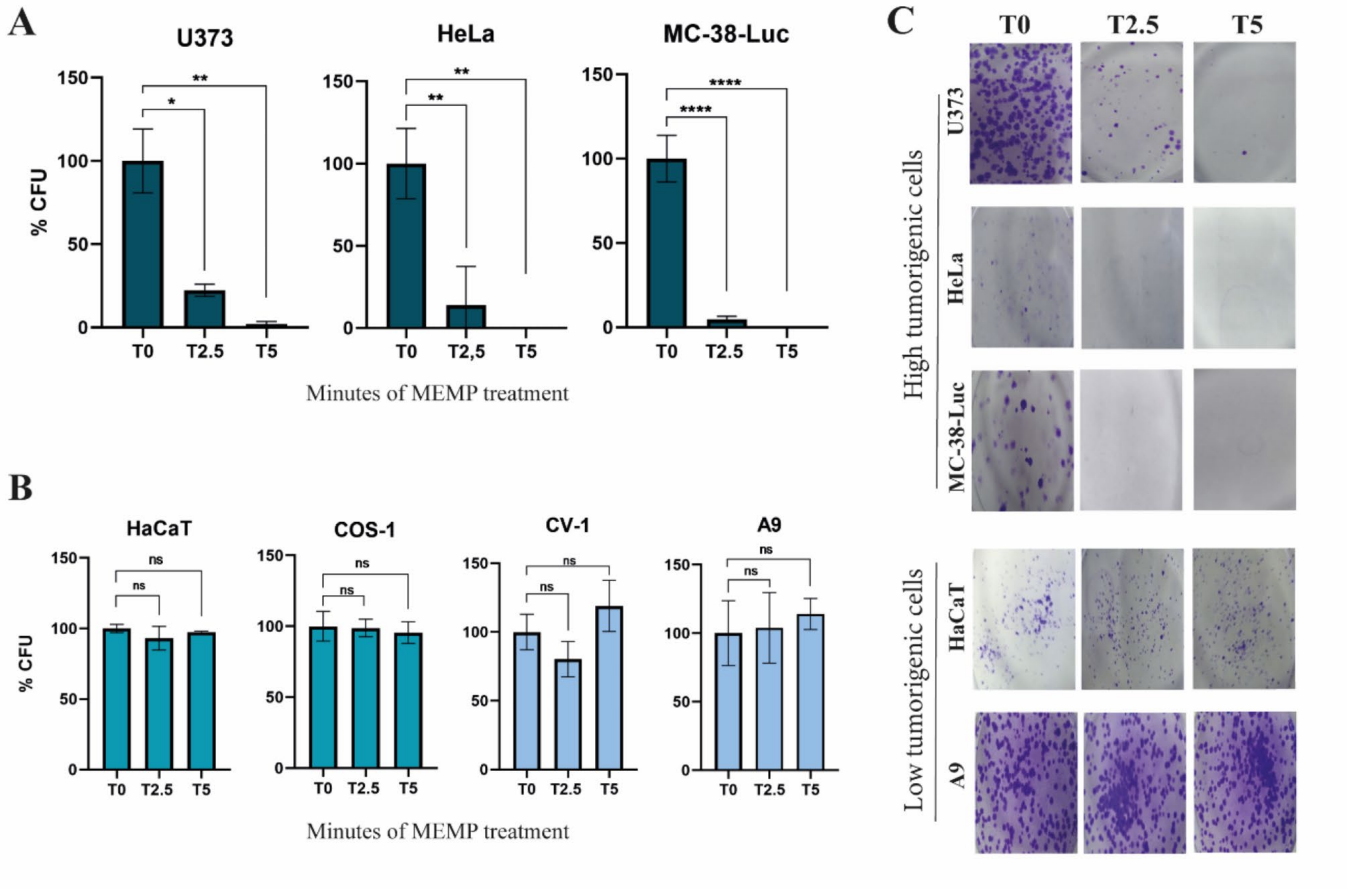
Differential inhibitory effect of MEMP treatments on cell lines assessed by colony forming ability. Control and modulated cell lines at the times indicated in the figure were seeded in triplicate at 10^3^ to 10^4^ cells onto P60 plates. The percentages of colonies formed in culture were calculated with respect to the number of colonies formed in the control (T0). (**A**) Percentage of colonies formed by U373, HeLa, and MC-38-Luc cells upon the MEMP treatments performed between 2.5 to 5 min. (**B**) Analysis of the inhibition of colony forming capacity caused by MEMP applied for 2.5 and 5 min to the HaCaT, COS-1, CV-1, and A9 cell lines. (**C**) Representative photographs showing the effect of MEMP treatment on colony formation by different cell lines. Colonies were fixed and stained with crystal violet two to three weeks after treatments. Data were statistically analyzed by using a one-way ANOVA with Dunnett’s test (*P < 0.05; **P < 0.01; ****P < 0.0001).

The efficacy of MEMP treatment was tested by colony formation assay, that is useful to determine tumor cell lines ability to form colonies after any kind of treatment. U373 MG, HeLa, and MC-38-Luc cancer cells were modulated for 2.5 and 5 min and their colony-forming ability was evaluated after two weeks (Fig. 2A-C). The obtained data supported the previous MTT result, with a progressive, modulation time-dependent, inhibition of colony-forming capacity. Thus, similarly to the viability assay, the HeLa cells seem to be a bit more susceptible than U373 cells, with no colony formation after 5 min of modulation, although at 2.5 min a slightly larger percentage of colonies was observed. The susceptibility of MC-38-Luc colony forming ability to MEMP (Fig. 2A–C) confirmed this adenocarcinoma cell line as the most sensitive to the treatment, as a very low percentage of colonies formed when modulated for 2.5 min and no colonies were detected at 5 min of treatment.

To assess the colony-forming behavior of poorly tumorigenic cell lines to the MEMP treatment, the HaCaT, COS-1, CV-1, and A9 cell lines were tested. Cells were modulated for 2.5 and 5 min and their colony-forming ability was scored 15 days afterwards. The analysis showed that all tested cell lines of low tumorigenicity were able to form colonies with no significant differences between non-modulated controls and 5 min modulated cells (Fig. 2B,C). Figure 2C illustrates representative results on colony forming capacity of the tested cell lines illustrating their distinct resistance to modulation. In summary, this test confirmed the preferable anti-proliferative effect of the MEMP treatment on malignant cells and therefore its therapeutic potential to be used in cancer treatment.

### Cytometric evaluation of MEMP modulation effect on cell viability

**Fig. 3:**
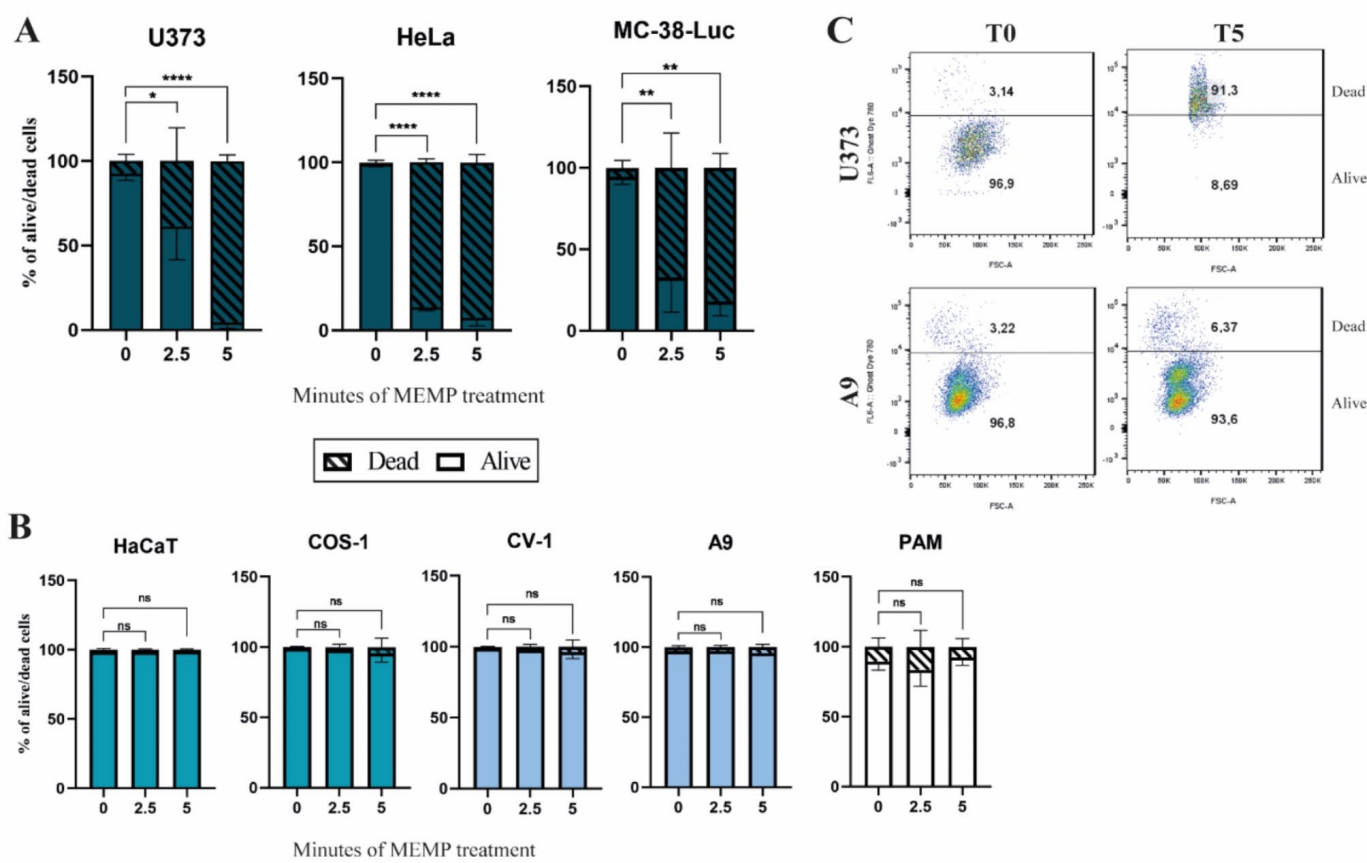
Determination of cell viability after MEMP treatment by cytometry. Cells modulated at times indicated in the figure were stained with the fixable Viability Dye Ghost Dye Red 780 (1 µg/mL) and fixed. Cells were then analyzed in a FACS Canto flow cytometer (BD Science) to determine the percentage of death and live cells. The figure shows representative data from three experiments on the percentages of live and dead cells after each MEMP modulation condition. Data were statistically analyzed by using a two-way ANOVA with Dunnett’s test (*P < 0.05; **P < 0.01; ****P < 0.0001). (**A**) Evaluation of MEMP effect on the viability of the U373, HeLa, and MC-38-Luc cancer cells. The experiment was repeated three times. (**B**) Determination of cell viability by FACS after the MEMP treatment in the HaCaT, CV-1, COS-1, A9 cell lines, and PAM. (**C**) Indicative photograph of dot plots showing the percentage of death and live U373 and A9 cells.

U373 and HeLa cells were evaluated by cytometry using the Ghost Dye 780, a marker of cell membrane functioning (see Materials and Methods), to investigate cell viability after the MEMP modulation procedure. Cells were tested at T0 (CTR), T2.5, and T5 (minutes of modulation). As Fig. 3 shows, the cytometric assay confirmed the progressive effect of the modulation on cell viability during the times of treatment on HeLa, MC-38-Luc, and U373 cells. The percentage of dead cells was higher in Hela and MC-38-Luc than in U373 cells, denoting some difference in their susceptibility to the modulation. In particular, the MEMP effect was especially high on MC-38-Luc cells as the T2.5 treatment did not show viable cells.

Subsequently, the effect of the treatment on several poorly tumorigenic cell lines was studied to gain a comparative view of susceptibility versus tumorigenicity. To address this, the percentage of death and live cells was evaluated on the poorly tumorigenic HaCaT, COS-1, CV-1, and A9 cell lines, as well as in PAM. This cytometric analysis showed that modulation did not significantly affect these cells (Fig. 3B). These results were consistent with the MTT and colony formation assay data described above and confirmed the resistance of the poorly tumorigenic cells to the MEMP treatment, as compared to the malignant cell lines tested.

### MEMP modulation induces cell cycle deregulation in human and mouse cancer cell lines

**Fig. 4:**
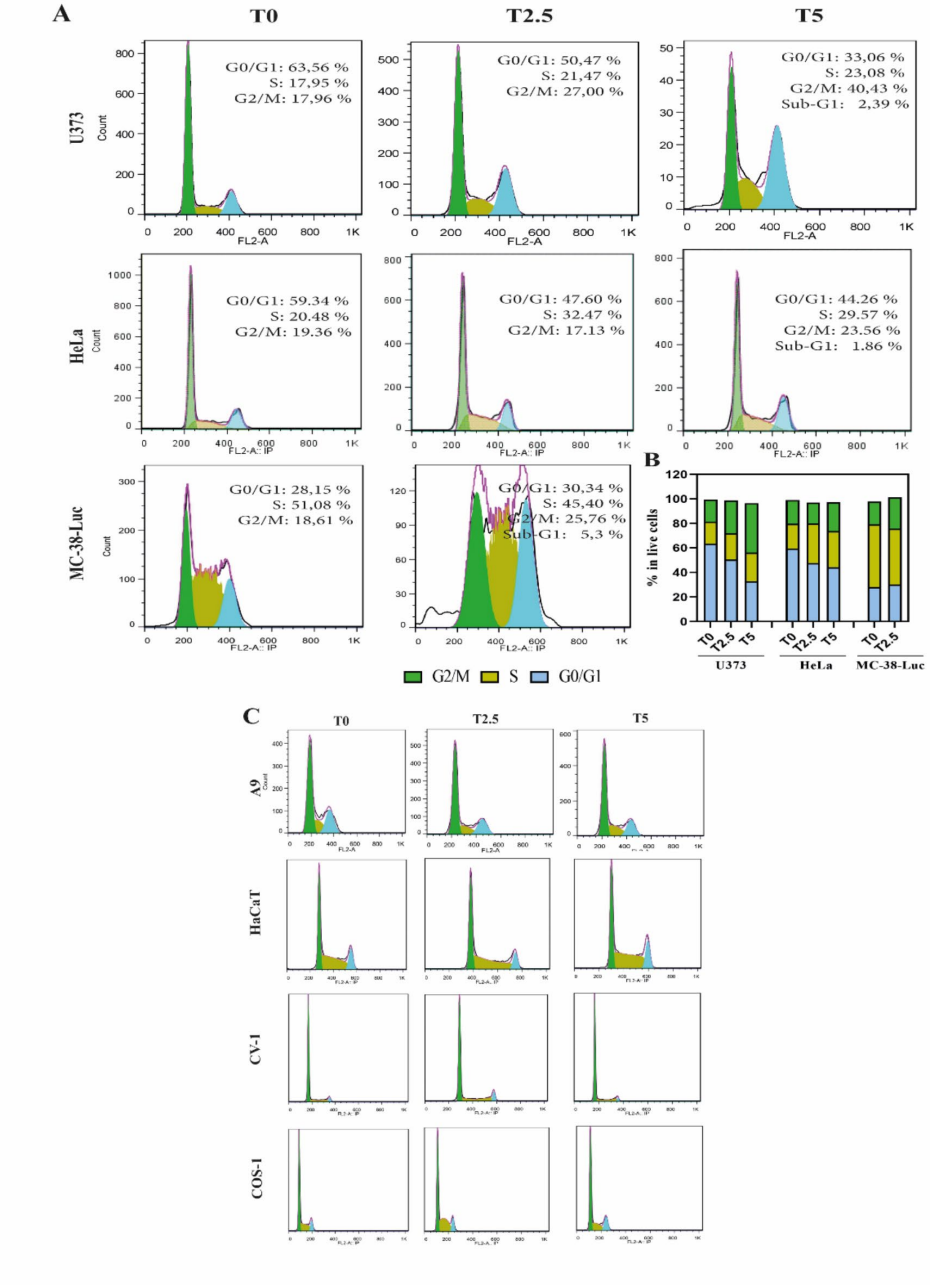
MEMP impact on cell cycle control. (**A**,**B**) Cell cycle distribution of malignant U373, HeLa and MC-38-Luc cell lines, and (**C**) low tumorigenic A9, HaCaT, CV-1 and COS-1 cell lines, after MEMP modulation treatments (). The profiles were obtained by FACS Calibur flow cytometry (BD Science), and cell cycle progression was based on DNA quantification. Cells were modulated for 5 min, then collected at 48 h afterward, fixed and stained with the PI/RNase buffer (BD Pharmingen).

In order to investigate the molecular effect of MEMP treatment on malignant and non-malignant cell lines, the cell cycle pattern was analyzed by flow cytometry. In particular, U373, HeLa and MC-38-Luc cancer cell lines were analyzed and compared with A9, HaCaT, CV-1, and COS-1 low tumorigenic cell lines. FACS analysis showed a progressive deregulation of cell cycle in all the malignant cells tested, with an increase of G2-phase proportional to the time of modulation (Fig. 4). In particular, in U373 cells (Fig. 4A,B) we found an important increase of G2-phase arrested cells after 2.5 and 5 min of MEMP treatment, with a progressive decrease of G1-phase. In addition, at T5, a further increase of sub-G1 phase was observed, suggesting apoptotic events in these conditions. Conversely, HeLa cells do not show a G2 increase after a 2.5 min MEMP treatment, although a moderate S increase and G1 decrease was noticed (Fig. 4A,B). Subsequently, upon a 5 min MEMP treatment, a G2 increase with a progressive G1 and S decrease, accompanied by a sub-G1 increase (1.86%), was registered, suggesting a gradual deregulation of the cell cycle that appears to occur more slowly than in the U373 cells. Regarding mouse colon adenocarcinoma MC-38-Luc cells, due to their high sensitivity to MEMP, we were able to analyze only cells modulated for 2.5 min, since after that time no intact cells could be collected. Thus, as shown in the panels of Fig. 4A,B, the modulation treatment produced a sharp cell cycle deregulation with a marked increase of G2-phase (25.76%) compared with T0 (no treatment), and a decrease of S-phase (45.4%). In addition, we observed an increase of sub-G1 phase, suggesting that cells are dying by apoptosis. In parallel, the low tumorigenic A9, HaCaT, CV-1, and COS-1 cells were also analyzed by FACS after 2.5 and 5 min of MEMP treatment. The analysis showed no significant changes, as compared to the non-modulated controls, in the cell cycle phases of all these poorly tumorigenic cell lines at any time of treatment (Fig. 4C). These results indicated that under the tested MEMP modulation conditions, the physiological control of cell cycle progression of the low tumorigenic cells was not affected.

### The MEMP treatment induces immediate actin cytoskeleton collapse of human glioblastoma cells

**Fig. 5:**
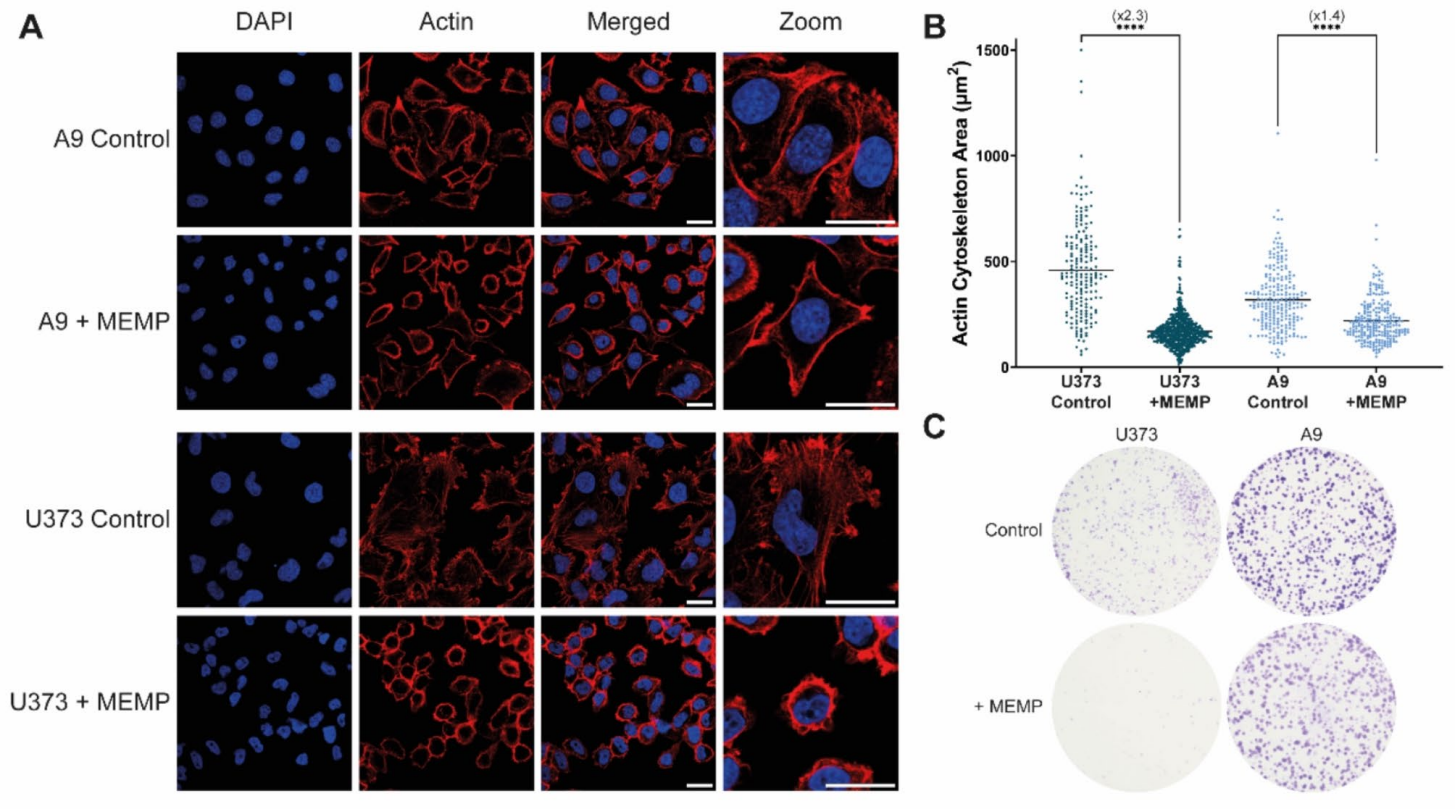
Evaluation of MEMP effects on cell shape and actin cytoskeleton organization. Cells were treated with MEMP for two minutes and immediately fixed for subsequent inspection by confocal microscopy with specific antibodies. (**A**) Fields of A9 and U373 cells stained with the indicated antibody and phalloidin and analyzed by confocal microscopy at 60x magnification. Scale bars, 20 μm. (**B**) Quantitative determination of the collapse of the actin cytoskeleton as determined by confocal microscopy (see Materials and Methods). Each dot represents a single cell (N, at least180 cells per condition). Statistics was performed using the Brown-Forsythe and Welch ANOVA and the Games-Howell´s multiple comparison tests. (**C**) Representative picture showing the colony forming ability of A9 and U373 cells at the MEMP regime used in A and B.

Attempting to identify a major cellular component that could be a target of the MEMP, we focused in the cytoskeleton, as this large and highly organized macromolecular entity is stabilized by polar charges and previous reports suggested affectation by electric pulses^13–15^. In our study, a possible direct and immediate effect of MEMP on cell shape and cytoskeleton was examined by confocal microscopy. To this aim, mouse A9 fibroblasts and human U373 glioblastoma cells were submitted to MEMP for a 2 min time treatment and immediately fixed in paraformaldehyde. Visual inspection by confocal IF microscopy showed that the actin cytoskeleton remained under a normal expanded configuration in the bulk of the MEMP-treated A9 cells (Fig. 5A, upper). In contrast, the actin cytoskeleton immediately collapsed around the nucleus in most treated human U373 cells (Fig. 5A, lower). A quantitative measurement of the effect of the MEMP on the area covered by the actin cytoskeleton (Fig. 5B) showed a significant 2,3-fold drastic collapse in the U373 cell population, whereas this ratio was only 1,4-fold in the A9 cells. Interestingly, the nuclear envelope was not significantly impaired by MEMP either in U373 or in A9 cells as judged by the confined DAPI staining of chromatin (Fig. 5A left and zoom panels). Of note, the precise time conditions of the MEMP used compromised the colony-forming ability in a cell type-dependent manner (Fig. 5C) as described above, suggesting that the population of A9 fibroblasts with partially collapsed actin cytoskeleton recovered nevertheless their proliferative capacity upon culturing to form colonies of normal sizes.

### MEMP treatment inhibits in vivo tumorigenicity of mouse colon cancer cells

**Fig. 6:**
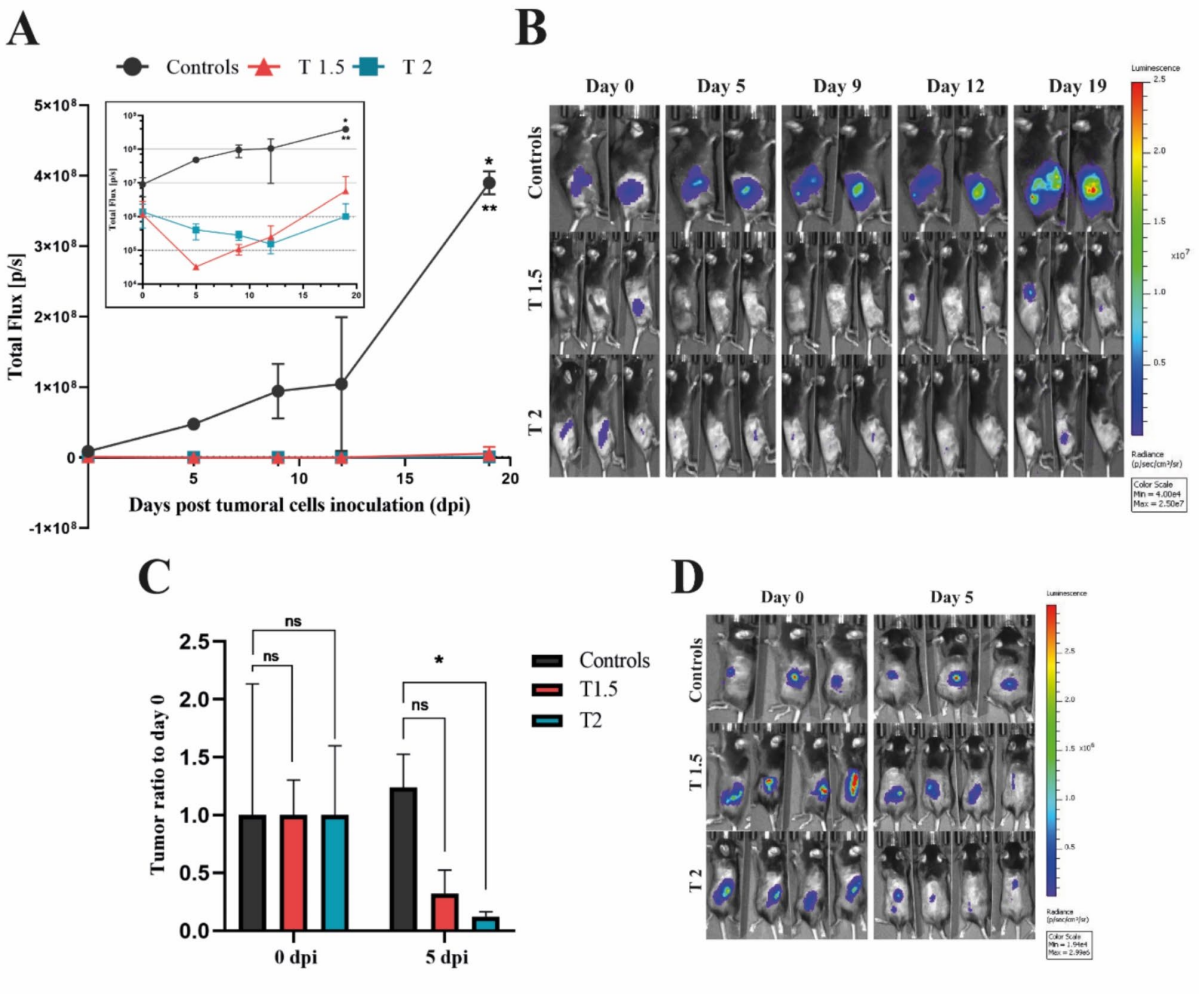
Evaluation of tumor forming capacity of modulated colon cancer cells in mice. Mouse MC-38-Luc adenocarcinoma cells were modulated by MEMP for 0, 1.5, and 2 min and then 0.5 × 10^6^ control and modulated cells were injected per C57BL/6 mouse. (**A**) In vivo imaging quantification of tumor bioluminescence from day 0 (30 min post-inoculation) until day 19 (sacrifice of untreated cells-inoculated mice) of the first experiment. *Inset*: the same graph in log10 scale. Data were statistically analysed using a two-way ANOVA with Tukey post-hoc test. (**B**) IVIS images from day 0 until 19 days post-inoculation (radiance p/sec/cm^2^/sr color scale Min = 4.00e4, Max = 2.50e7) of the first in vivo experiment. (**C**) In vivo tumor progression from day 0 to day 5 post-inoculation based on the tumoral ratio to day 0 of all mice of the second experiment. Statistic was performed using a two-way ANOVA with Dunnett’s test. (**D**) IVIS images at day 0 and day 5 post-inoculation (radiance p/sec/cm^2^/sr color scale Min = 1.94e4, Max = 2.99e6) of the second experiment.

To test the effect of MEMP on the tumorigenic capacity of cancer cells, Mc-38-Luc tumor cells growing in monolayers were modulated for 1.5–2 min in PBS, collected, and subsequently subcutaneously injected into immunocompetent C57BL/6 mice in parallel with control non-modulated cells. In order to respect animal welfare, a limited number of mice was used in two independent experiments. Tumor progression in all mice was monitored by bioluminescence in vivo imaging. Importantly, as shown in Fig. 6, all mice from the two experiments regardless the modulation treatment of the injected cells, showed the first day of xenografting high luciferase activity at the site of injection, denoting significant metabolic activity of the 1.5- and 2-min modulated cells. As the first experiment proceeded (Fig. 6A, B), untreated control cells formed tumors that showed the expected high growth rate characteristic of this in vivo tumor model. In contrast, mice inoculated with cells treated for 1.5 min, had no measurable tumors until day 9, followed by a restricted growth that only overcame the initial light emission by day 19 post-injection Moreover, mice injected with cells treated for 2 min did not show measurable tumor growth until day 19 post-injection (Fig. 6A), indicating that the tumor-forming capacity of most cells was impaired by the MEMP treatment. The second independent experiment (Fig. 6C, D) consistently showed suppressed tumor growth by 5 days in mice injected with modulated cells. Collectively, these two experiments demonstrated the capacity of MEMP regimes to abruptly suppress the in vivo tumorigenicity of colon adenocarcinoma cells.

### In vivo MEMP treatment is safety

To test the safety of the MEMP treatment, four C57BL/6 healthy mice were subjected to the modulation and health parameters evaluated over the time. Mice weighed before and after (72 h) the MEMP treatment reported no change in their weight (Table S1). As other health parameter, blood was collected at 72 h after MEMP treatment, and the number of counted leucocytes/mm^3^ fall into the range of number of leucocytes in normal mice and, importantly, this number was similar in modulated and control mice (Table S1), ruling out a bone marrow aplasia syndrome. These measurements were repeated in all animals at day 10th post-modulation, showing again no significant differences between treated and control animals (Table S2), confirming the absence of signs of anaplasia as well as the safety of the treatment. In parallel, other health parameters of the animals such as appetite, anxiety conditions, signs of pain, and symptoms, which could be associated with the modulation treatment, were recorded daily. In consistency, these data did not show any significant health difference between control and modulated mice, confirming the well-being of all animals. In summary, the obtained data demonstrate no important side effects of MEMP on animal welfare, supporting future applications in the treatment of developing tumors in vivo.

## Discussion

Several studies that refer to different electromagnetic fields used to cause effects on cancer cells and tumors^2,4,5,36–38^, as a decrease in cell proliferation^6^ and tumor remission in some cases^7^, have been reported. However, current research in this field does not clearly detail the exact mechanisms by which electromagnetic fields interact and impair cancer cell physiology. Other studies have referred to the different electrical characteristics of cancer cells, considering the electronegativity of some of their structures^9,39^, but to date, no scientific evidence supports the molecular mechanisms that differentiate the electronegativity of a cancer cell from a normal cell. In spite of these uncertainties, this field is currently being widely explored for potential therapies.

For the development of this study, we have considered a relationship of relative electronegativity with well-known characteristics of cancer cells, placing several basic physical science concepts in the context of molecular biology. It is known that in physics, relative electronegativity may be affected by diverse elements such as the effective nuclear charge, the size of the atoms, the electronic configuration, the inductive effects, the resonant effects, or the hybridization^40^. The electronic configuration itself may be altered in a controlled manner with electronic perturbations, what would provoke variations in the relative electronegativity of a biological structure such as a cancer cell. It should also be noted that when a more electronegative organic element is exposed to an electromagnetic field, the chemical bonds are more likely to be polarized, triggering a greater response of that element to the electromagnetic field, which may impair the molecular interactions and functions of the molecules themselves. This phenomenon may explain why a specific electronic perturbation has a significant impact on a more electronegative cancer but not on a non-tumorigenic cell.

In the context of the aberrant metabolism of cancer cells undergoing the Warburg effect^41^, relative electronegativity is caused by the accumulation of acid metabolites that increase the concentration of ionized hydrogen atoms (H⁺)^42^. Indeed, the biochemical processes that may impact the electronegativity of cancer cells are multiple and complex. Best known examples may be summarized as follows: (I) Abnormal expression of proteins and biomarkers, as the kinesin family members in breast cancer that influence cellular electronegativity through changes in microtubule dynamics and ionic charge distribution^43,44^, the PYCR2 and ADH1A in hepatitis B virus-related hepatocellular carcinoma altering cellular electronegativity^45^, and the NNMT in the tumor stroma regulating histone methylation and other transcriptional changes that are critical for phenotyping of cancer-associated fibroblasts (CAF), which can influence cellular cargo^46^; (II) Metabolic reprogramming including changes in glucose and other nutrient metabolism, as the regulation of serine metabolism and glycolysis through mTOR signaling indirectly affecting the electrical charge in pancreatic cancer; (III) Increased oxidative stress due to excessive production of reactive oxygen species (ROS), which influence the functionality and survival of the cancer cell^47,48^; (IV) Mechanisms related to cell signaling and the release of inflammatory mediators, which may have domains with different charges and electrical properties or can modify the activity of certain ion pumps and channels in the cell membrane^49,50^; (V) Changes in the composition of phospholipids and cholesterol of the cytoplasmic membrane in response to inflammation and cancerous transformation may modify its electrical properties and consequently also the electronegativity of the cell^51,52^; and (VI) Functional integrity of the mitochondrial and of endoplasmic reticulum membranes, as alterations may impact protein synthesis and folding or lead to programmed cell death^53,54^.

In this work, we have exposed a collection of mammalian cells with different origins and tumorigenic capacities (see Table 1) to MEMP applied at various time points. The applied parameters remain invariable because they are controlled externally by the modulator, which ensures their independence from the specific tumorigenicity of the cells. Tumorigenicity is tightly related to metabolic activity in a wide sense, including signaling activity, membrane transport, and the many other processes that as mentioned above are related to the electronegativity of the cancer cells^19,55^. It is therefore remarkable that our experimental data show a fair correlation between the degree of tumorigenicity and the sensitiveness of the tested cells to the MEMP (Figs. 1, 2, 3 and 4). A possible exception to this correlation was the COS-1 cell line, obtained by SV40 transformation of the CV-1 cells (see Table I) and expressing the tumorigenic T antigen that binds and perturbs the p53 and pRb and other cell major regulators^18^, which was not susceptible to the MEMP treatments (Figs. 1, 2, 3 and 4). Whether this result represents an inconsistency with the overall found correlation between tumorigenicity and MEMP effects is unclear since COS-1 tumorigenicity in mice remains to be described. Interestingly, our results may suggest that major tumor suppressors (p53 and pRB) and connected transformation processes may not drastically alter the cell metabolic pathways leading to electronegativity, a hypothesis deserving further investigation.

Importantly, our study uncovered a series of molecular mechanisms impacted by the MEMP regimes that may account for the compromised vital functions of the malignant cells. For example, a major finding was the capacity of MEMP to induce cell cycle deregulation in several highly tumorigenic cell lines, with a significant increase of the G2-phase. In particular, in the U373 human glioblastoma cells, the MEMP treatment was able to cause a G2-phase block, with a clear arrest of cells at the S-phase and an increase of sub-G1 phase, suggesting apoptotic events. MC-38-Luc colon cancer cells show also a cell cycle deregulation with an increase of sub-G1 phase (see Fig. 4), suggesting that these cells are dying by apoptosis as well.

Another important molecular target of the MEMP was the cytoskeleton. We found that this treatment induces an immediate abrupt collapse of the actin cytoskeletons in U373 glioblastoma, which occurs within a 2 min time period post-treatment, while in A9 mouse fibroblasts the actin cytoskeleton spread configuration remained much less affected (Fig. 5). The drastic and rapid actin cytoskeleton collapse induced by MEMP suggests that these highly organized macromolecular structures may be a primary target of the electromagnetic fields. However, the remarkable different response of the actin cytoskeleton in normal A9 and carcinogenic U373 cells to the MEMP treatments is a striking result. Although a comprehensive definitive explanation cannot yet be provided, it should be remembered that electrical potentials and bioelectrical signals regulate cell behavior and influence cellular processes such as migration^56^, in which cytoskeleton dynamics play a key role. Furthermore, the actin microfilaments play major roles as bionanowires capable of propagating ionic information^57^, and the electrical impulse along actin filaments are altered under pathological conditions^58^. Indeed, the bioelectric state of cancer cells is different from that of healthy cells, causing a disruption in the cellular signaling pathways^59^. Therefore, although the structure of the actin cytoskeleton may not show significant differences in its intrinsic polar charges between normal and cancer cells, the dysfunctional bioelectrical environment of cancer cells may indirectly impact their cytoskeleton organization, which would substantially influence their response to electromagnetic stimuli such as those used in this study.

Interestingly, actin cytoskeleton collapse may transmit apoptotic and death signaling toward cytosolic mediators as described^60,61^, accounting for the viability decay and cell cycle deregulation observed in the modulated malignant cells (Figs. 1, 2, 3 and 4), although further research would be need to confirm this possibility. In the perspective of the time at which each molecular process was MEMP-affected and detected in malignant cells, we show that cytoskeleton collapse occurs within minutes, cell permeability and cell cycle perturbation in 20–24 h, proliferative clonogenic capacity in two weeks days, and tumorigenicity in vivo in three weeks. Our study illustrates the effective cascade of perturbations that may be caused on the physiology of malignant cells through time-limited MEMP regimes.

Finally, this study also shows the capacity of the MEMP to drastically suppress the tumorigenicity of malignant colon adenocarcinoma cells when treated for 2 min prior to xenograft transplantation in immunocompetent mice (Fig. 6). Although the treatment was performed in vitro prior to xenografting, the prospective value of the assay relies on the capacity of the treated cells to maintain metabolic activity upon injection, as determined by luciferase expression. However, the treated cells absolutely lost their malignancy as tumor growth was halted and metabolic activity disappeared two weeks post-injection. This assay show promises for the treatment of pre-established tumors with the non-invasive MEMP technology, either alone or in combination with other clinically used anti-cancer therapies.

## Electronic supplementary material

Below is the link to the electronic supplementary material.

Supplementary Material 1

Supplementary Material 2

## Data Availability

All data generated or analysed during this study are included in this published article.
