# Supplementary material for: Assessment of molecular modulation by multifrequency electromagnetic pulses to preferably eradicate tumorigenic cells
Source: Sci Rep. 2024 Dec 3;14:30150. doi: 10.1038/s41598-024-81171-x (PMC11615363; doi:10.1038/s41598-024-81171-x)
Supplement: Supplementary file 1 — Supplementary Material 1 [file 41598_2024_81171_MOESM1_ESM.pdf]

Assessment of Molecular Modulation by Multifrequency Electromagnetic Pulses to Preferably Eradicate Tumorigenic Cells

Roberta Piredda[1], Luis G. Rodríguez Martínez[2], Konstantinos Stamatakis[1,4], Jorge Martinez-Ortega[1], Alejandro López Ferráz[3], José M. Almendral[1*], and Yolanda Revilla[1*]

1. Centro de Biología Molecular Severo Ochoa (CSIC-UAM). Universidad Autónoma de Madrid. 28049 Cantoblanco, Madrid, Spain.
2. Universidad de Sancti Spíritus, Cuba
3. Universidad de La Laguna, Santa Cruz de Tenerife, Spain.
4. IRYCIS, Madrid, Spain.

Table S1. Mice weight and White blood cells number before and 72h post- MEMP treatment

Mice	Weight before treatment (g)	Weight after treatment (g)	Leucocytes/mm3
F1	18,75	19	6500
F2	17,15	17	12200
M1	24,5	25	15550
M2	25,7	25,9	15100

2 females (F1 and F2) and two males (M1 and M2) were subjected to a MEMP treatment and their weight and white blood cells determined 72h afterwards.
