# Supplementary material for: Assessment of molecular modulation by multifrequency electromagnetic pulses to preferably eradicate tumorigenic cells
Source: Sci Rep. 2024 Dec 3;14:30150. doi: 10.1038/s41598-024-81171-x (PMC11615363; doi:10.1038/s41598-024-81171-x)
Supplement: Supplementary file 2 — Supplementary Material 2 [file 41598_2024_81171_MOESM2_ESM.pdf]

Assessment of Molecular Modulation by Multifrequency Electromagnetic Pulses to Preferably Eradicate Tumorigenic Cells

Roberta Piredda[1], Luis G. Rodríguez Martínez[2], Konstantinos Stamatakis[1,4], Jorge Martinez-Ortega[1], Alejandro López Ferráz[3], José M. Almendral[1*], and Yolanda Revilla[1*]

1. Centro de Biología Molecular Severo Ochoa (CSIC-UAM). Universidad Autónoma de Madrid. 28049 Cantoblanco, Madrid, Spain.
2. Universidad de Sancti Spíritus, Cuba
3. Universidad de La Laguna, Santa Cruz de Tenerife, Spain.
4. IRYCIS, Madrid, Spain.

Table S2. Mice weight and leucocytes number 10 days post- MEMP treatment

Mice	Weight (g)	Leucocytes/mm3
F1	18,68	8536
F2	17,29	10043
F3 (control)	20,13	4050
F4 (control)	21,18	9615
F5 (control)	20	11970
M1	25,70	10904
M2	26,79	5561
M3 (control)	27,9	11970
M4 (control)	26,77	10856
M5 (control)	26,73	8925

Weight and white blood cells in control female (F3-F5) and male (M3-M5) mice and in female (F1, F2) and male (M1, M2) mice ten days after a MEMP modulation.
